# Norovirus-Specific Memory T Cell Responses in Adult Human Donors

**DOI:** 10.3389/fmicb.2016.01570

**Published:** 2016-10-03

**Authors:** Maria Malm, Kirsi Tamminen, Timo Vesikari, Vesna Blazevic

**Affiliations:** Vaccine Research Center, University of TampereTampere, Finland

**Keywords:** norovirus, T cell epitope, PBMC, VLP, peptide pools, cellular immunity, ELISPOT IFN-gamma

## Abstract

Norovirus (NoV) is a leading cause of acute gastroenteritis in people of all ages worldwide. NoV-specific serum antibodies which block the binding of NoV virus-like particles (VLPs) to the cell receptors have been thoroughly investigated. In contrast, only a few publications are available on the NoV capsid VP1 protein-specific T cell responses in humans naturally infected with the virus. Freshly isolated peripheral blood mononuclear cells of eight healthy adult human donors previously exposed to NoV were stimulated with purified VLPs derived from NoV GII.4-1999, GII.4-2012 (Sydney), and GI.3, and IFN-γ production was measured by an ELISPOT assay. In addition, 76 overlapping synthetic peptides spanning the entire 539-amino acid sequence of GII.4 VP1 were pooled into two-dimensional matrices and used to identify putative T cell epitopes. Seven of the eight subjects produced IFN-γ in response to the peptides and five subjects produced IFN-γ in response to the VLPs of the same origin. In general, stronger T cell responses were induced with the peptides in each donor compared to the VLPs. A CD8^+^ T cell epitope in the shell domain of the VP1 (^134^SPSQVTMFPHIIVDVRQL^151^) was identified in two subjects, both having human leukocyte antigen (HLA)-A^∗^02:01 allele. To our knowledge, this is the first report using synthetic peptides to study NoV-specific T cell responses in human subjects and identify T cell epitopes.

## Introduction

Norovirus (NoV) is recognized as a leading cause of acute gastroenteritis in children and adults worldwide (Hall et al., 2016). According to a recently published WHO report, NoVs caused 120 million cases of diarrheal diseases, more than any other foodborne or infectious agent (Havelaar et al., 2015). These non-enveloped RNA viruses belonging to *Caliciviridae* family are highly infectious and cause frequent outbreaks that can be serious to individuals with underlying conditions, the elderly, and young children (Hall et al., 2016). Currently, there is no cure or preventive vaccine available against NoV gastroenteritis.

The ∼7.6 kb-long single-stranded positive-sense RNA genome has three ORFs that encode for the replicase polyprotein (ORF-1), major VP1 capsid protein (ORF-2), and minor capsid protein VP2 (ORF-3; Prasad et al., 1999). NoVs are genetically classified into seven genogroups (GI to GVII), which are further divided into genotypes based on capsid VP1 amino acid sequence diversity (Kroneman et al., 2013; Vinje, 2015). NoV genotypes associated with human infections belong primarily to GI (9 genotypes) and GII (22 genotypes), with a >50% divergence of the VP1 at amino acid-level (Zheng et al., 2006; Kroneman et al., 2013). Variants of the most efficiently evolving GII.4 genotype, with approximately >95% homology in VP1 sequences, have predominated over the last two decades (Bok et al., 2009). VP1 consists of a shell (S) domain, a hinge region, and a protruding (P) domain, the latter of which is further divided into the P1 and extremely variable P2 subdomains; they contain sites important for host cell interaction (Cao et al., 2007). Ninety dimeric VP1 proteins form the outer layer of the icosahedral virus particle, which can vary in size from ∼27 to 40 nm, depending on the genotype (Vinje, 2015). VLPs are self-assembled by the recombinant capsid protein VP1. As a high-yield NoV cell culture system is still elusive (Jones et al., 2015), NoV VLPs are essential for structural and immunogenicity studies, and they are considered promising vaccine candidates (Blazevic et al., 2011; Atmar et al., 2015).

Norovirus infections occur early in life (Hinkula et al., 1995; Nurminen et al., 2011) and antibody seroprevalence reaches almost 100% by adulthood (Jing et al., 2000; Carmona-Vicente et al., 2015). Multiple sequential infections by genetically distinct NoV strains have been reported to occur frequently, especially in young children (Saito et al., 2014; Blazevic et al., 2015b). According to most studies, the duration of protection in adults is relatively short and limited to genetically similar virus strains (Wyatt et al., 1974; Johnson et al., 1990; Simmons et al., 2013). There is controversy over the correlates of protective NoV immunity and the length and specificity of the protection, which is complicated by the genetic diversity of NoVs (Siebenga et al., 2009; Vinje, 2015) and differences in the pre-existing NoV immunity of humans (Lindesmith et al., 2010, 2015). Furthermore, the distinctive expression pattern of polymorphic HBGAs affects individual susceptibility to NoV infections (Lindesmith et al., 2013). HBGAs, found, e.g., on the respiratory and gastrointestinal tract surface epithelia and in bodily secretions, are recognized and bound by NoV particles in a genotype-specific manner (Uusi-Kerttula et al., 2014) and putatively serve as the initiation site for NoV infection (Hutson et al., 2002; Huang et al., 2003; Cao et al., 2007). Serum IgG antibody titers blocking NoV VLP binding to HBGA have been most frequently associated with protection from NoV infection and disease (Reeck et al., 2010; Nurminen et al., 2011; Malm et al., 2014; Atmar et al., 2015). Humoral immunity to homotypic strains is efficiently elicited (Malm et al., 2014), and broadly cross-reactive NoV-specific IgG antibodies are found after NoV exposure (Rockx et al., 2005; Lindesmith et al., 2010; Malm et al., 2014). However, the induction of cross-protective antibodies capable of blocking NoV VLP-HBGA binding interaction have been observed only to a certain degree between the different genotypes of the GII or GI genogroups, but not across the genogroups (Reeck et al., 2010; Atmar et al., 2015; Blazevic et al., 2015c). Additionally, mucosal IgA responses have been associated with the blocking activity of NoV-HBGA binding (Tamminen et al., 2016) and protection from infection and NoV gastroenteritis (Lindesmith et al., 2003; Ramani et al., 2015).

While most NoV immunity studies have focused on humoral immune responses (Ramani et al., 2016), only three studies have gathered information on the role of cell-mediated immunity (CMI), especially T cells in NoV infection (Tacket et al., 2003; Lindesmith et al., 2005, 2010). Control of the pathogenesis with several viral infections, such as HIV-1 (Rosenberg et al., 1997; Blazevic et al., 2000; Seder et al., 2008), the influenza virus (Wilkinson et al., 2012; La Gruta and Turner, 2014), and the human papilloma virus (Stanley, 2006), is known to be dependent on T cell-mediated immunity, especially CTLs. Therefore, T cells are also likely to play an important role in NoV clearance. Following NoV infection/challenge, IFN-γ secretion by CD4^+^ T cells has been analyzed only by ELISAs using NoV VLPs as antigens, and the results indicated the generation of NoV-specific memory T cell responses (Lindesmith et al., 2005, 2010). Furthermore, the essential role of T cells in controlling NoV infections has been provided by studies with murine NoV (Chachu et al., 2008; Tomov et al., 2013).

In the present study, we employed ELISPOT IFN-γ and ICS assays to study *ex vivo* NoV-specific T cell responses in adult human subjects for the first time. We used 18-mer synthetic peptides derived from the NoV GII.4 sequence organized into matrix peptide pools to identify NoV-specific T cell epitopes.

## Materials and Methods

### Human Blood Donors

Venous blood samples (30 ml) were obtained from eight healthy adult (age range 26–56, **Table 1**) volunteers (laboratory personnel) with no symptoms of acute gastroenteritis in the past year. Additional blood of two volunteers (Donors 2 and 4) was collected for subsequent ICS experiments. Informed consent was obtained from each volunteer prior to the sample collection in accordance with the Declaration of Helsinki. No approval by an ethical committee was needed. PBMCs were isolated from the heparinized blood samples within 6 h of collection by Ficoll-Paque PLUS (GE Healthcare, Little Chalfont, UK) density gradient and suspended in cell medium (CM) containing RPMI 1640 with Glutamax^®^ and HEPES (Gibco^TM^ by Thermo Fisher Scientific, Waltham, USA) supplemented with 10 μg/ml Gentamicin (Gibco^TM^) and 10% fetal bovine serum (FBS, Sigma–Aldrich, St. Louis, MO, USA). Plasma was collected from each donor’s blood sample and stored at -20°C until further use. Human leukocyte antigen (HLA) typing was done by HLA locus-specific amplification by PCR and the subsequent probing of the product by sequence-specific oligonucleotide probe (PCR-SSOP, ProImmune Ltd., Oxford, UK).

**Table 1:** T cell interferon gamma (IFN-γ) responses to NoV antigens.

Donor #	Age (years)	CM	GII.4-99 VLP	GII.4 SYD VLP	GI.3 VLP	GII.4-99 peptide pool^a^	CEF peptide pool	OVA peptide
1	38	13^b^	**80**	**63**	**128**	**90**	**355**	15
2	35	0	10	15	8	**268**	**225**	0
3	56	3	**50**	**53**	**90**	**80**	**1345**	3
4	36	0	23	**50**	**75**	**640**	**1390**	3
5	47	0	18	23	20	28	**763**	3
6	44	3	**60**	33	38	**95**	**1698**	15
7	26	8	**55**	**50**	**170**	**140**	**1248**	8
8	45	10	**50**	35	40	**58**	**325**	18

*^a^GII.4-99 complete peptide pool containing 76 single peptides. ^b^Mean spot forming cell (SFC) values per 10^*6*^ PBMCs of parallel wells in ELISPOT IFN-γ assay. Positive SFC (≥50 SFC) are bolded. VLP, virus-like particle; CM, cell media; CEF, cytomegalovirus, Epstein–Barr Virus and Influenza virus; OVA, ovalbumin; SFC, spot-forming cells; SEM, standard error of the mean.*

### Recombinant Proteins

**FIGURE 1:**
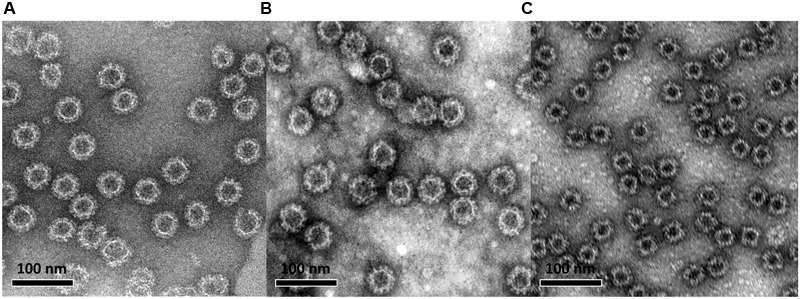
**Electron microscopy image of baculovirus-insect cell system-produced and sucrose gradient-purified norovirus GII.4-99 **(A)**, GII.4 Sydney **(B)**, and GI.3 **(C)** virus-like particles (VLPs).** VLPs were negatively stained with 3% uranyl acetate (pH 4.5) and the preparations were examined using an FEI Tecnai F12 electron microscope at a magnification of 23,000×.

Norovirus VP1 genes of the ancestor GII.4-1999 (GII.4-99, reference strain accession no: AF080551), the most recent GII.4 Sydney (SYD, accession no: AFV08795.1), and the representative genotype of GI genogroup, GI.3-2002 (accession no: AF414403), were cloned into pFastBac1 vector (Invitrogen, Carlsbad, CA, USA) and transfected in *Spodoptera frugiperda* (Sf)9 insect cells (Invitrogen) as described in detail elsewhere (Blazevic et al., 2011). The amplified BV stocks were used (multiple of infection, MOI = 1) for the production of NoV VP1 proteins in Sf9 cells which self-assembled into VLPs (**Figure 1**). The harvested VLPs were purified by two consecutive discontinuous sucrose gradient ultracentrifugations as previously described (Huhti et al., 2010). The residual sucrose was removed from the VLP preparations by dialysis against a PBS followed by a concentration step with Amicon Ultra-30 filtering units (Merc Millipore, Billerica, MA, USA). The protein concentrations of the purified VLP stocks were determined by BCA Protein Assay (Pierce^TM^ by Thermo Fisher Scientific). The purity, morphology, and *in vitro* and *in vivo* antigenicity of the VLPs were analyzed by sodium dodecyl sulfate polyacrylamide gel electrophoresis, electron microscopy (EM, **Figure 1**), ELISA, and mouse immunization experiments as earlier published by our laboratory (Huhti et al., 2010; Blazevic et al., 2011; Uusi-Kerttula et al., 2014; Malm et al., 2015).

### Synthetic Peptides

Seventy-six individual synthetic peptides designated 99-1 to 99-76 representing the entire 539 amino acid (aa) sequence of GII.4-99 NoV VP1 were synthetized (Synpeptide Co. Ltd, Shanghai, China) as 18-mers with adjacent peptides overlapping by 11 aa. The purity exceeded 70%, as determined by high pressure liquid chromatography. Each lyophilized peptide was dissolved in dimethyl sulfoxide (DMSO, Sigma–Aldrich) and further diluted in sterile PBS (Lonza, Basel, Switzerland) for use in the assays. The amount of DMSO never exceeded 0.01% in the final medium. A complete peptide pool (containing all 76 peptides) and 18 two-dimensional matrix pools (M1–M18) were made from individual peptides as previously described (Wilkinson et al., 2012). The first dimension included nine pools (M1–M9), the second dimension also included nine pools (M10–M18), and each pool contained eight or nine individual peptides so that each peptide was present in two different matrix pools. A CEF peptide pool (CTL Europe GmbH) containing 32 viral T cell epitopes from the human cytomegalovirus virus, the Epstein–Barr virus, and the influenza virus was used as a positive control peptide pool (Currier et al., 2002) in the ELISPOT assays. A murine T cell epitope (McFarland et al., 1999) derived from OVA (323–339, Invivogen, San Diego, CA, USA) was used as an irrelevant negative control peptide in the assays.

### Norovirus-Specific Serology Assay

An ELISA was utilized to detect the NoV GII.4-99-, GII.4 SYD-, and GI.3-specific IgG antibodies in the human samples as previously described (Malm et al., 2014). In brief, plasma diluted serially starting at 1:100 was added to NoV VLP-coated (25 ng/well) half-area 96-well plates (Corning Inc., Corning, NY, USA) and incubated for 1h at +37°C. One known NoV-positive and -negative human serum was added to each plate as controls. Blank wells lacking a sample were added as background controls. The bound antibodies were detected by HRP conjugated anti-human IgG (Novex^®^ by Thermo Fisher Scientific) reacting with o-phenylenediamine dihydrochloride substrate (OPD, Sigma-Aldrich). The OD values of each well were measured in a microplate reader (Victor^2^ 1420, Perkin Elmer, Waltham, MA, USA). The results are expressed as the end-point titers of the individual plasma determined as the highest titer giving an OD value above the set cut-off value (mean OD for negative control wells + 3 × SD and at least 0.100 OD) after background subtraction.

### ELISPOT IFN-γ Assay

Fresh PBMCs of the eight donors were assayed in an ELISPOT assay for IFN-γ production upon stimulation with NoV VLPs (GII.4-99, GII.4 SYD, and GI.3) or synthetic GII.4-99 peptides used as a complete peptide pool, peptide matrices, and in subsequent experiments as single peptides. Ninety-six-well nitrocellulose filter plates (Millipore) were coated at +4°C (18–72 h) with anti-human IFN-γ capture antibody (Mabtech) at a concentration of 5 μg/ml. After washing with sterile PBS, the plates were blocked for 2–3 h with CM containing 10% FBS. The antigens were added to the plates: VLPs at final concentrations of 0.5 and 5 μg/ml and GII.4-99 peptide pools at 2 μg/ml. The dilutions of the final peptide pools used in the assay were determined from earlier titration experiments. The single peptides were used at 0.5–4 μg/ml final concentration. Positive control (CEF-pool, at 1.5 μg/ml) and negative control (OVA 323–339, at 2 μg/ml) peptides and CM only (background control) were tested on each plate. Fresh PBMCs were added at 0.2 × 10^6^ cells/well and the plates were incubated for 44 h at +37°C and 5% CO_2_. After discarding the cells, the plates were washed and biotinylated anti-human IFN-γ antibody (Mabtech) was added at 2 μg/ml. The plates were incubated for 3 h at RT followed by 1 h incubation with 1:500 diluted streptavidin-HRP (BD, Trenton, NJ, USA). The spots were developed for 7.5 min with Vector Nova Red substrate (Vector Labs, Burlingame, CA, USA) in the dark and the reaction was stopped with tap water. The plates were air-dried prior to automated spot counting by an ImmunoSpot Series II analyzer (CTL Europe, Leinfelden-Echterdingen, Germany). The results are expressed as mean SFC/10^6^ PBMCs of the duplicate wells. The results were considered positive if the number of SFC per well was ≥50 spots/10^6^ PBMCs and twice above the background control (CM only wells). SFC in the background control wells never exceeded 15 SFC/10^6^ PBMCs.

### Intracellular Cytokine Staining (ICS)

An ICS assay was employed to measure IFN-γ, TNF-γ and IL-2 cytokine production, and to determine the T cell phenotype responsible for cytokine secretion. Fresh PBMCs of two donors were stimulated (1 × 10^6^ cells/condition) with 2 μg/ml 99–20 single peptide or with 1 μg/ml SEB (Sigma) in the presence of 1 μg/ml CD28 and 1 μg/ml CD49d costimulatory antibodies (BD Biosciences, San Jose, CA, USA) for 2 h at 37°C. As a control, PBMCs were incubated in CM only supplemented with the costimulatory antibodies. The protein transport inhibitor brefeldin A (GolgiPlug, BD Biosciences, San Jose, CA, USA) at a concentration of 10 μg/ml was added and the incubation was continued for 16 h. After the stimulation, cells were treated with EDTA for 15 min to arrest activation and remove adherent cells, and washed with FACS Stain buffer. Non-specific binding was blocked by incubating the cells for 10 min with Human BD Fc Block. Cell surface markers CD3 and CD8 were stained before the permeabilization of the cells by incubating the cells with monoclonal antibodies against human CD3 (clone UCHT1, FITC conjugate) and CD8 (clone RPA-T8, PerCP-Cy5.5 conjugate) in 50 μl Stain buffer for 30 min on ice in the dark. Cells were washed twice with Stain buffer and treated with BD Fixation/Permeabilization solution for 20 min according to the manufacturer’s instructions. Fixed and permeabilized cells were washed twice with BD Perm/Wash buffer before intracellular staining with the mixture of IFN-γ (clone 4S.B3) phycoerythrin (PE)-Cy7-conjugate, IL-2 (clone MQ1-17H12) phycoerythrin-conjugate, and TNF-α (clone MAb11) APC-conjugate in 50 μl Perm/Wash buffer for 30 min on ice in the dark. After the ICS incubation period, PBMCs were washed twice with Perm/Wash buffer and resuspended in Staining Buffer for acquisition and analysis. All reagents used for ICS were purchased from BD Pharmingen (San Jose, CA, USA).

### Flow Cytometric Analysis

**FIGURE 2:**
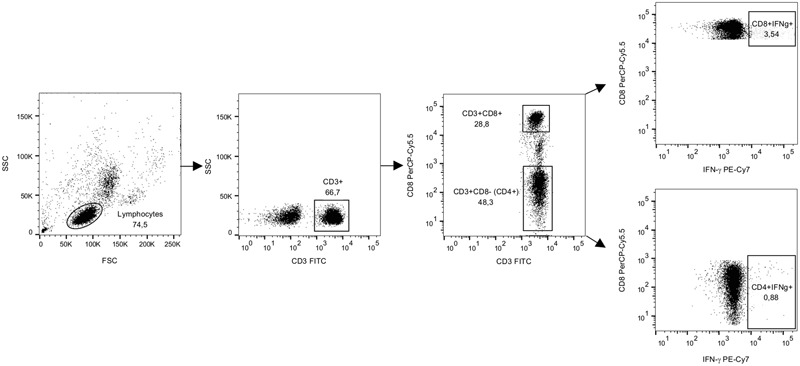
**The gating strategy for flow cytometry analysis from representative data.** PBMCs from a donor were stimulated with SEB for 16 h in the presence of brefeldin A and then stained for IFN-γ and lineage markers CD3 and CD8. A forward and side scatter was used for gating the lymphocyte population. CD3^+^ lymphocyte population was further gated to CD8^+^ and CD8^-^ populations and IFN-γ staining was analyzed for both populations as previously described by others (Meddows-Taylor et al., 2007). Dot plots show gated populations and events as percentages of the parent population.

Samples were acquired using a 2-laser FACS CantoII flow cytometer (BD) and FACSDiva Software V 6.1.3 (Becton Dickinson, Heidelberg, Germany) within 2 h of staining with fluorescent antibodies. PMT voltages were adjusted with the unstained, fixed PBMC sample. At least 70,000 CD3^+^ cells were acquired per sample. The gates were designed in the negative control sample with consideration of the marker downregulation from the stimulated samples. The gating strategy is shown in **Figure 2**. The final analysis plots of IFN-γ IL-2, and TNF-α were gated on CD3^+^CD8^+^ and CD3^+^CD8^-^ populations. The data analysis was performed using FlowJo software version 10.1 (Tree Star, San Carlos, CA, USA). Cytokine responses were defined as positive if the percentages of cytokine-positive cells stimulated with the antigens were at least two times higher and >0.05% above the cytokine positive cells in the background (CM; Ferrari et al., 2004).

### Statistical Analyses

Fisher’s exact test was used to compare the differences in the IgG end-point titers. The Mann–Whitney *U*-test was used to assess the differences between NoV VLP- and GII.4-99 peptide pool-induced SFC/10^6^ PBMCs in the ELISPOT. Spearman’s rank correlation coefficient was used to examine the correlation between the SFC/10^6^ PBMCs and the antibody end-point titer. Statistical analyses were performed using IBM SPSS Statistics (SPSS, Chicago, IL, USA) version 23.0. Statistical significance was defined as *p* < 0.05. All hypothesis tests were two-tailed.

## Results

### Antibody Responses

**FIGURE 3:**
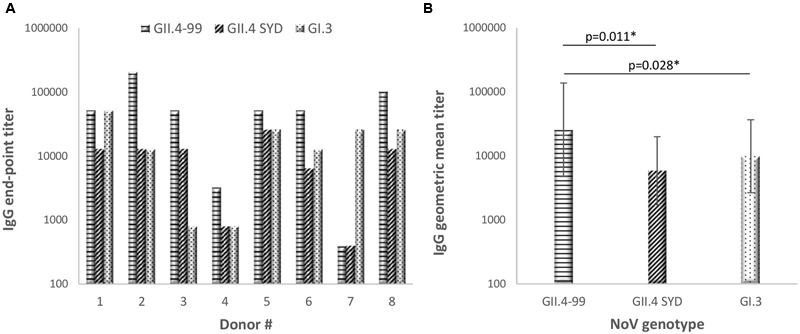
**Norovirus genotype-specific immunoglobulin G (IgG) antibody titers.** The plasma of eight donors was used to determine individual end-point titer **(A)** and geometric mean titers (GMTs) with 95% confidence intervals **(B)** against NoV GII.4-1999, GII.4 Sydney, and GI.3 VLPs in an enzyme-linked immunosorbent assay (ELISA). Statistical significance was determined by Fisher’s exact test, and a *p*-value ≤ 0.05 was considered to be statistically significant (^∗^).

NoV GII.4-99, GII.4 SYD, and GI.3 VLPs were used as antigens in an ELISA to test plasma IgG antibody binding to native, conformational antigenic determinants. The results showed that all eight donors had NoV-specific antibodies (**Figure 3A**), indicating previous NoV exposure, but the magnitude of the genotype-specific responses varied among the donors. All donors had antibody response to GII.4-99, and the end-point titers to this genotype were the highest of the three VLPs tested (**Figure 3B**, *p* < 0.05).

### T Cell Response to NoV VLPs and Capsid Peptide Pool

An ELISPOT assay was utilized to detect *ex vivo* IFN-γ release from human PBMCs after *in vitro* stimulation for 44 h with NoV VLPs (GII.4-99, GII.4 SYD, and GI.3) or a complete peptide pool derived from GII.4-99 capsid VP1. None of the donors responded to stimulation with 0.5 μg/ml VLPs (data not shown). **Table 1** shows that when using a high concentration (5 μg/ml) of the VLPs, six of the eight donors responded to at least one VLP, and three donors produced IFN-γ in response to all VLPs tested. Interestingly, all but one donor (#5) responded to the GII.4-99 peptide pool, but only five responded to GII.4-99 VLPs (**Table 1**). Furthermore, the number of IFN-γ-secreting cells in response to the peptide pool was higher (mean 175 ± 71.2 SFC/10^6^ cells) than the response to the corresponding GII.4-99 VLPs (mean 43 ± 8.5 SFC/10^6^ cells; *p* < 0.05). However, there was a difference in the magnitude of these responses in each individual and high variation among the donors was seen, especially with the peptide pool stimulation (58–640 mean SFC/10^6^ PBMCs). Two donors (#2 and #4), who were the best responders to the GII.4-99 peptide pool, had no response to the VLPs of the same origin. Only one donor (#5) did not respond either to the VLP or peptide pool stimulation. PBMCs of all eight donors produced IFN-γ after the CEF peptide pool stimulation (>200 SFC/10^6^ PBMCs) and none responded to the negative OVA control peptide (<20 SFC/10^6^ PBMCs).

### Correlation of T Cell and Antibody Responses

**FIGURE 4:**
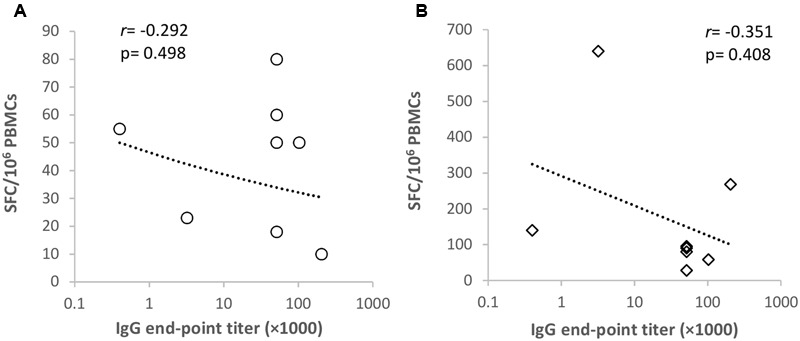
**Correlation of GII.4-1999-specific antibody and T cell responses.** Spearman’s rank correlation (*r*) was determined between the norovirus GII.4-1999-specific antibody titer and ELISPOT IFN-γ spot-forming cell (SFC)/10^6^ peripheral blood mononuclear cells (PMBCs) in response to GII.4-99 VLPs **(A)** and a complete capsid peptide pool **(B)** stimulation.

Next, we determined whether NoV VLP-specific antibody titers correlated with the corresponding genotype T cell responses detected in the ELISPOT IFN-γ assays (**Figure 4**). No correlation (*p* > 0.05) was observed between GII.4-99 antibody titers and IFN-γ-producing PBMC numbers after the VLP (**Figure 4A**) or complete peptide pool (**Figure 4B**) stimulation. Similarly, no correlation was detected between antibody titers and IFN-γ-secreting cell numbers against GII.4 SYD (*r* = -0.268, *p* = 0.521) or GI.3 (*r* = 0.328, *p* = 0.618) VLPs (data not shown).

### Mapping of T Cell Epitopes with Matrix Peptide Pools

**FIGURE 5:**
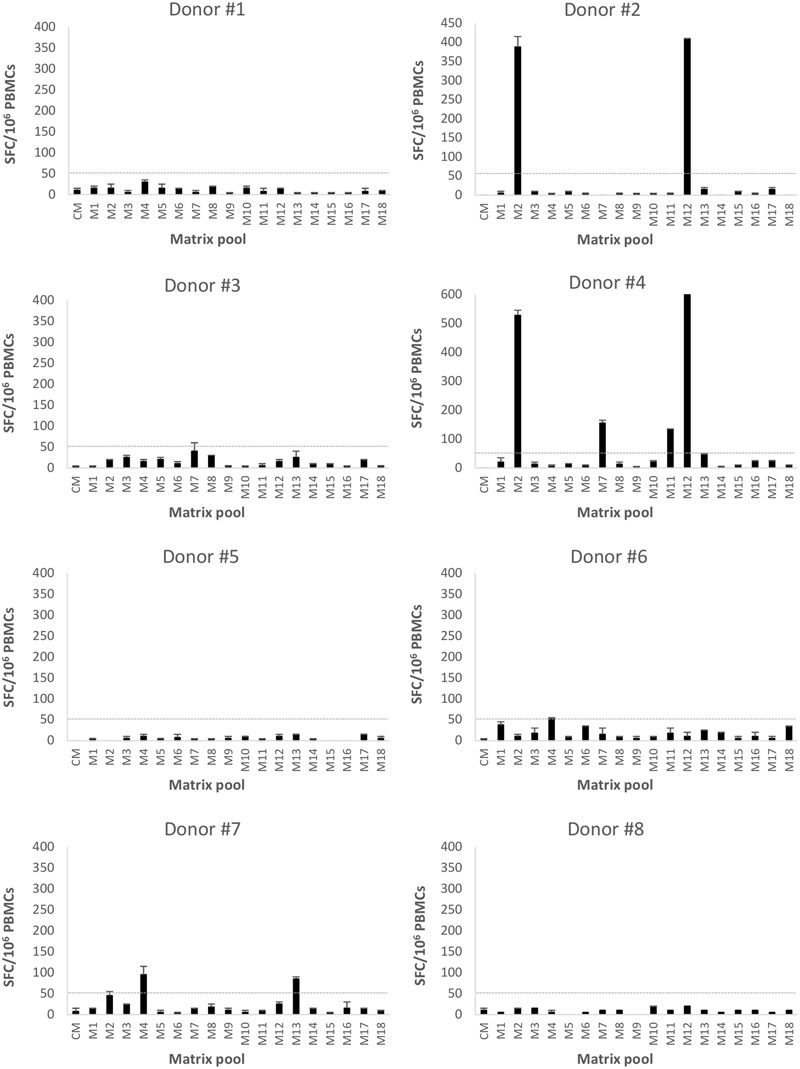
**Enzyme-linked immunosorbent spot interferon gamma (IFN-γ) responses to GII.4-1999 matrix peptide pool stimulation.** Peripheral blood mononuclear cells (PBMCs) of eight donors (#1-8) were stimulated with 76 18-mer GII.4-99 capsid-derived single peptides organized into 18 matrix pools (M1–M18) or left unstimulated (CM, culture media). Shown are the mean spot-forming cells (SFC)/10^6^ PBMCs of two replicate wells with the standard errors of the mean. The cut-off line (dotted line) represents the positive SFC/10^6^ value of >50 SFC/10^6^ cells.

Seventy-six 18-mer overlapping peptides spanning the entire GII.4-99 VP1 were used to identify NoV-specific T cell epitopes. PBMCs of each donor were assayed for IFN-γ production in an ELISPOT assay in response to the peptides organized into 18 matrix pools (M1–M18). Only three of the eight donors (donors #2, #4, and #7) responded to at least two matrix pools (**Figure 5**), and the same donors also had the strongest response to the complete GII.4-99 peptide pool (**Table 1**). The strongest peptide-specific IFN-γ response was toward matrix pools M2 and M12 in donors #2 and #4. Donor #4 also responded moderately to matrix pools M7 and M11, as did donor #7 to M4 and M13 (**Figure 5**).

For each donor responding to the matrix pools, stimulatory peptides contained in the pools were deconvoluted from a two-dimensional matrix system, where each peptide appeared only once in each dimension. Three single peptides were identified as putative T cell epitopes: 99-16 peptide (aa 106-123) included in the M7 and M11 pools (donor #4), 99-20 peptide (aa 134-151) included in the M2 and M12 pools (donors #2 and #4), and 99-31 (aa 211-228) in the M4 and M13 pools (donor #7).

### T Cell Restriction of the Epitopes

**FIGURE 6:**
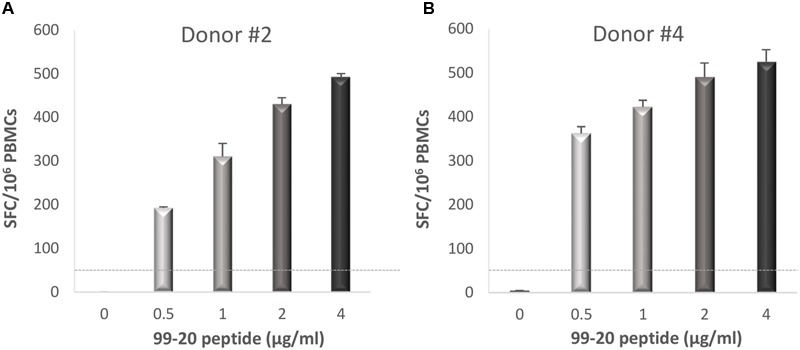
**Enzyme-linked immunosorbent spot IFN-γ responses toward NoV GII.4-1999 T cell epitope 99-20.** Fresh peripheral blood mononuclear cells (PBMCs) of donors #2 **(A)** and #4 **(B)** were stimulated with an increasing concentration (0–4 μg/ml) of peptide 99-20 containing the putative NoV-specific T cell epitope. Shown are the spot-forming cells (SFC)/10^6^ PBMCs of two replicate wells with standard errors of the mean. The cut-off line (dotted line) represents the positive SFC/10^6^ value of >50 SFC/10^6^ cells.

In the three donors with the T cell epitopes deconvoluted from a two-dimensional matrix system, newly isolated PBMCs were stimulated with the single peptides 99-16, 99-20, and 99-31 in the second ELISPOT IFN-γ assay. Only peptide 99-20 (^134^SPSQVTMFPHIIVDVRQL^151^) induced strong IFN-γ production in donors #2 (**Figure 6A**) and #4 (**Figure 6B**), who originally responded strongly to the M2 and M12 matrix pools (**Figure 5** and data not shown).

**FIGURE 7:**
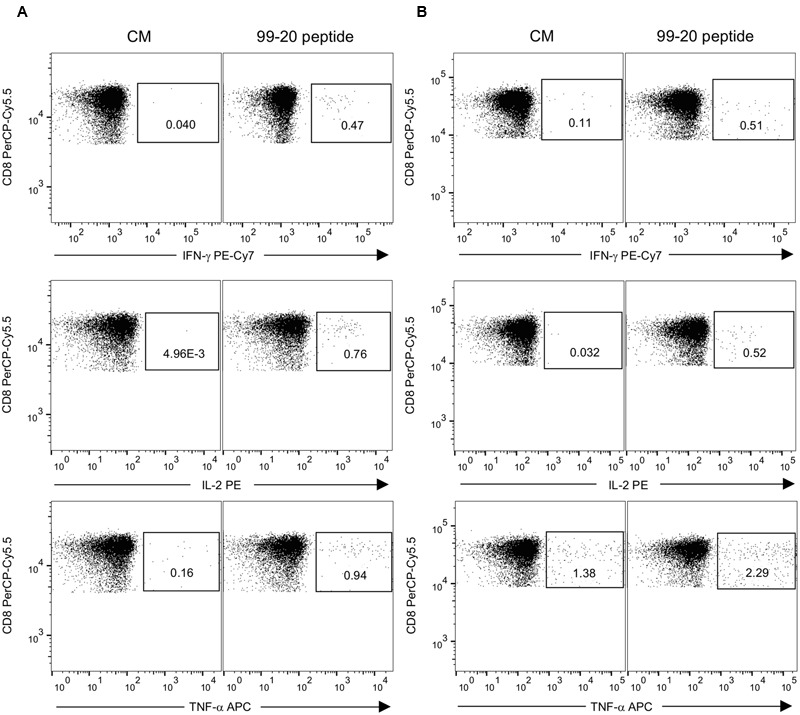
**Expression of IFN-γ, IL-2, and TNF-α by intracellular staining after stimulation with NoV GII.4-1999 peptide epitope 99-20.** Peripheral blood mononuclear cells (PBMCs) of donors #2 having A^∗^01:01/^∗^02:01; B^∗^08:01/^∗^56:01; C^∗^01:02/^∗^07:01 HLA class I phenotype alleles **(A)** and #4 having A^∗^02:01/^∗^32:01; B^∗^35:01/^∗^40:02; C^∗^03:04/^∗^04:01 HLA class I phenotype alleles **(B)** were stimulated with 2 μg/ml of 99-20 single peptide for 16 h and the cytokine response was measured by flow cytometry. PBMCs in culture media only (CM) (left panel) and peptide-stimulated PBMCs (right panel) were gated to CD3^+^CD8^+^ T cells and the percentages of IFN-γ, IL-2, or TNF-α cytokine-expressing cells are indicated.

Peptide 99-20 was further tested by ICS to identify the T cell subset responsible for IFN-γ production in donors #2 and #4 determined by the ELISPOT assay. Additionally, IL-2 and TNF-α cytokine secretion were simultaneously analyzed. Fresh PBMCs were stimulated with the peptide 99-20 or SEB as a positive control antigen. The unstimulated PBMCs (CM) sample served as a background control. The percentages of cytokine-producing cells were determined within the CD3^+^CD8^+^ and CD3^+^CD8^-^ (CD4^+^) T cell populations. Peptide 99-20 induced the expression of all three cytokines by CD3^+^CD8^+^ T cells for both donors (**Figure 7**), identifying the epitope as CD8^+^ T cell-restricted. These two donors were further typed for the HLA-A, -B, and -C alleles in order to identify possible common HLA class I restricting molecule binding to the peptide epitope 99-20. The typing results showed that both donors carry HLA-A^∗^02:01 allele.

Further analysis of the cells responding to peptide 99-20 by IFN-γ production (gating on CD3^+^CD8^+^IFN-γ^+^ lymphocytes) revealed that ∼74% of these populations in both donors were also simultaneously expressing IL-2 and TNF-α cytokines. No positive responses were found in the CD4^+^ T cell gate for any of the cytokines after the 99-20 peptide stimulation (data not shown). Both the CD3^+^CD8^+^ and CD3^+^CD8^-^ T cell populations of each donor were positive for all cytokines after SEB stimulation, confirming the good viability and functionality of the cells and a successful staining protocol (**Figure 2** and data not shown).

## Discussion

Very limited published information is available on natural T cell immunity in NoV-infected humans (Tacket et al., 2003; Lindesmith et al., 2005, 2010). CD4^+^ Th and CD8^+^ CTLs are important in protection from infection by different viruses, including influenza (La Gruta and Turner, 2014; Lartey et al., 2015), HIV (Rosenberg et al., 1997; Blazevic et al., 2000), HPV (Stanley, 2006), and the Epstein–Barr virus (Pepperl et al., 1998). Protective immunity to NoV might also be partially dependent on the activation of T cell immunity. Murine NoV establishes persistent infection in the absence of functional T cells, supporting the importance of T cell-mediated immunity in NoV infection (Wobus et al., 2006; Chachu et al., 2008; Tomov et al., 2013).

We studied antibody and T cell-mediated pre-existing immune responses in eight healthy adult donors. Each donor had a positive serum IgG antibody titer against all NoV VLPs tested, indicating previous exposures to the NoV (Jing et al., 2000; Nurminen et al., 2011; Carmona-Vicente et al., 2015). Two NoV GII.4 VLPs, derived from an ancestor GII.4-99 and the most recent GII.4 SYD variants, were chosen as antigens, as GII.4 has been the dominating NoV genotype for over two decades (Bull and White, 2011; Eden et al., 2014). GI.3 VLPs were chosen as a representative genotype of the GI genogroup contributing to a minority of NoV infections in humans (Matthews et al., 2012). The strongest antibody response in seven of the eight donors was detected toward GII.4-99 VLPs, suggesting that most of the donors have been exposed to GII.4-99 or antigenically closely related variants in the past. As these donors have likely not experienced infection with the recently circulating GII.4 SYD, the antibody responses to this genotype were significantly lower, probably consisting of cross-reactive antibodies raised to the earlier circulating variants of the GII.4 genotype (Rockx et al., 2005; Malm et al., 2015).

Different methods are employed to measure T cell responses in natural infections and vaccination studies. ELISPOT and ICS detecting IFN-γ secretion from PBMCs induced by synthetic peptides and/or recombinant whole proteins are the most commonly used (Schmittel et al., 2001; Currier et al., 2002; Kutscher et al., 2008; Streeck et al., 2009; Freer and Rindi, 2013; Kutscher et al., 2013; Lartey et al., 2015). In comparison to ICS, ELISPOT is highly sensitive but does not identify the cell type responsible for the cytokine production (Tassignon et al., 2005; Tobery et al., 2006). Therefore, using ICS to complement results from an ELISPOT assay is a worthwhile and generally used practice (Freer and Rindi, 2013; Lartey et al., 2015). To study T cell-mediated immunity, we investigated *ex vivo* IFN-γ production from freshly isolated PBMCs in response to stimulation with NoV VLPs and GII.4-99 synthetic peptides by both ELISPOT IFN-γ and ICS. We detected a much higher frequency and magnitude of T cell responses using an ELISPOT IFN-γ assay in previously exposed adult human donors toward synthetic peptides (pooled and individual) than the whole protein assembled in the VLPs. All but one donor responded to the complete GII.4-99 peptide pool, but only five responded to GII.4-99 VLPs. Furthermore, two donors had a strong CD8^+^ T cell response to GII.4-99-specific peptide 99-20, but no response to the corresponding VLPs, as shown by the ELISPOT IFN-γ.

The greater response to the peptides than VLPs detected here is not surprising, as the 15-20-mer synthetic peptides added exogenously to the PBMCs circumvent the need for antigen processing by directly binding to HLA classes I and II molecules and stimulating both CD4^+^ and CD8^+^ T cells efficiently (Blazevic et al., 1995; Kern et al., 2000; Kaufhold et al., 2005). In contrast, protein antigens require processing into smaller peptide fragments by antigen-presenting cells before being presented for T cell recognition, preferentially stimulating CD4^+^ T cells through HLA class II molecules. The ELISPOT assay employing peptides usually requires overnight incubation (Schmittel et al., 2001; Kaufhold et al., 2005). Therefore, we used a 44 h assay to ensure processing of the VLPs and the binding of the peptide fragments to HLA molecules. Comparison of the assays performed at 20 or 44 h showed that NoV peptide-induced responses were comparable but the responses to the VLPs were slightly improved by a longer incubation period (data not shown). The absence of NoV seronegative samples implies problems in setting the cut-off for true positives and warrants further studies with the samples where the T cell response is absent, such as cord blood samples.

The 18-mer epitope 99-20 (^134^SPSQVTMFPHIIVDVRQL^151^), which induced strong IFN-γ, IL-2, and TNF-α production by CD8^+^ T cells in two donors, may be restricted to an identical HLA class I molecule shared between these two subjects or may contain overlapping epitopes restricted by different HLA class I molecules. Indeed, the HLA typing of the two donors identified the HLA-A^∗^02:01 as a possible restriction allele as both subjects carry this molecule. To fully describe the minimal epitope sequence and to confirm its restriction, future work is necessary. As the adjacent overlapping peptides (99-19 and 99-21, respectively) did not elicit IFN-γ responses (data not shown), the minimal T cell epitope is probably not contained in the overlapping 11 aa peptide segments. As optimal CD8^+^ T cell epitopes are 9 aa long (Kiecker et al., 2004; Murphy et al., 2007), mapping studies using peptides of a shorter length are required to identify the minimal epitope. Congruently, peptide binding predictions using the IEDB analysis resource and ANN (Nielsen et al., 2003; Lundegaard et al., 2008) and SMM (Peters and Sette, 2005) tools showed that the 10 aa sequence ^139^TMFPHIIVDV^148^, contained in the 18-mer peptide 99-20, has a high affinity binding (IC50 < 100 nM) to the HLA-A^∗^02:01 allele. Simultaneous production of these cytokines by NoV-specific CD8^+^ T cells indicates the generation of multifunctional memory T cells after natural infection. It was previously shown that human central memory CD8^+^ T cells – but not effector memory T cells – produce IFN-γ and IL-2 in response to virus epitopes (Mallard et al., 2004). These multifunctional T cells have been shown to be highly relevant for conferring protection from infections such as influenza (Seder et al., 2008; La Gruta and Turner, 2014; Lartey et al., 2015).

The 99-20 epitope is situated in the S domain of the capsid VP1 and is highly conserved between distant GII.4 variants. There is only 1 aa change in the sequence between the GII.4-99 and GII.4 SYD (^145^I to ^145^V). The 18 aa-long peptide sequence is highly specific to the GII.4 genotype and differs by five aa from other GII NoVs, including the GII.1, GII.2, GII.3, GII.12, and GII.17 genotypes; the aa at positions 136 (S), 138 (V), and 144 (I) are variable in all aligned GII strains.

In this study, no correlation was found between pre-existing NoV-specific antibody levels and T cell responses, however, the small sample size makes it difficult to interpret the absence of correlation. In contrast to the antibody responses, our results indicate that natural T cell immunity to NoV is quite weak. Congruently, limited information published thus far (Tacket et al., 2003; Lindesmith et al., 2010) has shown virtually undetectable baseline levels of NoV-specific T cell responses prior to challenge or immunization. The mapping studies performed here to detect the production of IFN-γ to synthetic peptides by the PBMCs of adult donors exposed to NoV in the past revealed a limited number of dominant T cell stimulatory epitopes in the NoV GII.4 capsid protein. Three putative T cell epitopes were identified using matrix pools, located in the S domain (peptide 99-16, aa 106-123 and peptide 99-20, aa 134-151) and at the junction of the S and P1 domains (peptide 99-31, aa 211-228) of the NoV capsid, but only one epitope was confirmed with the use of single peptides in the ELISPOT assay. Intramuscular immunization of mice of a single haplotype (H-2^d^) with GII.4-99 VLPs induced responses to seven peptide epitopes located alongside the capsid VP1 (unpublished observation), which is more than seen in the eight NoV-exposed human donors. Studies on recently exposed individuals or young children are needed to elucidate if NoV-specific T cell response differs after recent infection. It also remains to be seen if the NoV vaccination would induce novel epitopes to broaden the T cell responses in humans or/and increase the frequency of already existing NoV-specific T cells. Additionally, gut-associated lymphoid tissue needs to be investigated to determine T cell responses in the gut, which may be different from the peripheral blood T cells.

Short-lived antibody responses to NoV described previously (Johnson et al., 1990; Simmons et al., 2013; Blazevic et al., 2015a), together with a restricted memory T cell response to NoV described here, may be responsible for repeated NoV infections over an individual’s life time. Blocking (neutralizing) antibodies are the most recognized correlate of protection against NoV infection, and their activity is measured in current clinical trials with VLP-based vaccine candidates (Lindesmith et al., 2015; Ramani et al., 2016). Protection against NoV probably reflects the sum of various immune responses, including antibody and T cell responses. Therefore, measuring both arms of the adaptive immunity should be undertaken in clinical trials. More studies on NoV-specific T cell responses are needed to determine their role in protection from infection.

This article describes for the first time an ELISPOT IFN-γ method using synthetic peptides to measure NoV-specific T cell responses from freshly isolated PBMCs. In addition, we identified the first human NoV-specific CD8^+^ T cell epitope (^134^SPSQVTMFPHIIVDVRQL^151^) using this approach.

## Author Contributions

MM and KT, equal contribution, shared first authorship. Sample acquisition, processing and laboratory analysis (ELISA, ELISPOT, ICS and flow cytometry). Data acquisition, analysis and interpretation. Writing the manuscript. Final approval of the version to be submitted. MM only: corresponding author during the manuscript submission only. TV, the head of Vaccine research center. Critical revision of article for important intellectual content. Final approval of the version to be submitted. VB, the head of the laboratory. Corresponding author in the final published article. Conception and designing the study, data interpretation, drafting and writing the article, critical revision of article for important intellectual content. Final approval of the version to be submitted.

## Conflict of Interest Statement

The authors declare that the research was conducted in the absence of any commercial or financial relationships that could be construed as a potential conflict of interest.

## Abbreviations

APC: allophycocyanin
BV: baculovirus
CEF: cytomegalovirus, Epstein–Barr Virus and Influenza virus
CTL: cytotoxic T lymphocyte
ELISA: enzyme-linked immunosorbent assays
ELISPOT: enzyme-linked immunosorbent spot
FITC: fluorescein isothiocyanate
HBGA: histo-blood group antigen
HIV-1: human immunodeficiency virus-1
HLA: human leukocyte antigen
HRP: horseradish peroxidase
ICS: intracellular cytokine staining
IFN-γ: interferon gamma
IL-2: interleukin-2
NoV: Norovirus
OD: optical density
ORF: open reading frame
OVA: ovalbumin
PBMCs: peripheral blood mononuclear cells
PBS: phosphate buffered saline
PE: phycoerythrin
SEB: staphylococcal enterotoxin B
SFC: spot-forming cells
SYD: Sydney
Th: T helper
TNF-α: tumor necrosis factor alpha
VLP: virus-like particle
